# The Ethics of Overlapping Relationships in Rural and Remote Healthcare. A Narrative Review

**DOI:** 10.1007/s11673-023-10243-w

**Published:** 2023-03-28

**Authors:** Rafael Thomas Osik Szumer, Mark Arnold

**Affiliations:** School of Rural Health (Dubbo/Orange), Faculty of Medicine and Health, The University of Sydney, Dubbo, NSW Australia

**Keywords:** Health, Ethics, Rural, Remote, Dual relationship, Professional boundaries

## Abstract

It is presently unclear whether a distinct “rural ethics” of navigating professional boundaries exists, and if so, what theoretical approaches may assist practitioners to manage overlapping relationships. To be effective clinicians while concurrently partaking in community life, practitioners must develop and maintain safe, ethical, and sustainable therapeutic relationships in rural and remote healthcare. A narrative review was conducted identifying a significant body of qualitative and theoretical literature which explores the pervasiveness of dual relationships for practitioners working in rural and remote healthcare. Rather than viewing dual relationships as ethically unacceptable, much contemporary work focusses on the lived experiences of healthcare workers and explores what approaches may be available that both protect the therapeutic relationship while recognizing the unique nature of rural and remote healthcare practice. We conclude that practitioners must have a means of operating within a contextually informed ethics of professional boundaries. Drawing on pre-existing work, one schema is proposed that could form the basis for further engagement through interactive teaching sessions, professional development, mentoring, or guidelines.

## Introduction

The lived experiences of rural and remote healthcare practitioners are often vastly different from their urban colleagues. Where urban clinicians usually reside in geographically and culturally distinct worlds to those of their patients, rural clinicians share the same streets, schools, shops, and services with those they treat (Cook and Hoas 2019a). Rural healthcare practice is embedded in an environment where overlapping relationships are inevitable, expected, and often valued (Campbell and Gordon 2003).

The terms dual, multiple, and overlapping relationships are all used to describe the situation where a professional has two or more relationships with the same person with whom they engage in the course of their professional practice (Crowden 2008b). In health professional ethics literature, these relationships are often defined as either (a); boundary crossings: relatively benign transgressions of the traditional prohibition on relationships beyond the singular doctor–patient relationship, or (b); boundary violations: exploitative relationships that are harmful and conflict with the therapeutic relationship (Scopelliti et al. 2004). Overlapping relationships have traditionally been categorized as ethically problematic (Endacott et al. 2006), due to the imperative to protect the patient from harm, while remaining objective and dispassionate. Professionals are cautioned that boundary crossings may be a slippery slope toward potential boundary violations, and professional ethics is conventionally understood to require a clear separation between a treating practitioner’s clinical and personal worlds (Galletly 2004).

However, in recent decades the view that a treating practitioner must only have a singular relationship with their patient has been challenged as urban-centric in its ethical approach (Simpson and McDonald 2017) and in its ignorance of the realities of rural clinical practice (Gingerich et al. 2021). It has been posited that professional boundaries and their management in a rural context may be recast in a manner that recognizes their pervasiveness (Thomas, Boxall, .and et al 2014). The starting point of this contextual approach is to acknowledge the inevitability of overlapping relationships in rural healthcare, and then to ensure that they do not compromise the therapeutic relationship (Campbell and Gordon 2003). Various approaches to the management of overlapping relationships in rural practice have been proposed, most of which involve critical reflection on the part of the practitioner (Brownlee et al. 2019).

## Aims

This narrative review seeks to answer the following research questions:

i) Does the existing literature identify a distinct “rural ethics” of navigating professional boundaries?;
ii) What themes emerge from the literature in relation to negotiating overlapping relationships in rural practice; and
iii) Are there any approaches that may assist practitioners to manage professional boundaries in rural and remote healthcare identified in the literature?

In answering the above, this paper provides a schema to assist practitioners to navigate overlapping relationships and thereby develop and maintain ethical and sustainable therapeutic relationships in rural practice.

The impetus for the paper arose from the authors’ experience of living and working in rural and remote communities across Australia and the discussions between them that followed. In particular, since it is recognized that social isolation influences practitioners’ long-term retention in the rural workforce (Cosgrave et al. 2019) the authors wished to identify approaches that would mitigate the potential for social isolation. Finally, the authors recognize that a proportion of healthcare is provided by non-resident fly-in, fly-out or drive-in, drive-out practitioners, with which comes many other ethical challenges that are not the subject of this paper.

## Methodology

In order to accurately survey the contemporaneous landscape of professional boundaries in rural healthcare, the first author undertook a broad literature search and made a subjective decision for inclusion based on the following criteria:

i) Published in English between 2000–2021 to ensure contemporaneity;
ii) Qualitative study, narrative or systematic review, or commentary related to rural healthcare practice and in particular the existence or management of overlapping boundaries;
iii) a. Full text, available via the following academic databases: Medline, Embase, CINAHL, Scopus, and Informit; b. Grey literature was accessed via Google;
iv) a. MeSH terms used: Rural Population / Hospital, Rural [ethics]/ Ethics, Professional/ Ethics, Medical;
v) b. Search terms used included: rural, remote, boundary, boundaries, professional, ethics, multiple, dual, overlapping;
vi) Second pass searches were made through forward and backwards citation.
vii) The exclusion criteria of non-health related disciplines including teaching and law was applied. Disciplines fitting within health including social work had pertinent papers included; and
viii) Non-Australian publications were included following an assessment of relevance for Australian healthcare practitioners or those practicing in similar jurisdictions and comparable health systems in rural settings. This included that the paper was from a country with a health system broadly similar to Australia’s and that the content was relevant for the aims of this study.

Following a first pass search, approximately 180 papers were identified with further papers subsequently identified via second pass searches. Following removal of duplicates and papers deemed not relevant according to the criteria set out above, eighty-five documents formed the basis of this review. These were made up of six codes of conduct/ health reports, two books and seventy-seven papers made up of original research, review articles, perspectives, and commentary.

## Findings

The analysis identified five major themes in the literature:

1. The Reality of Overlapping Relationships in Rural and Remote Healthcare Practice

In rural environments, it is almost inevitable that a health practitioner’s everyday relationships will cross-over with their practice (Campbell and Gordon 2003). These relationships may range from the incidental such as being served in the shop by a patient, to the more involved such as engaging patients to undertake work or having a friend seek treatment (Cleret 2005). For some professionals such as Aboriginal Health Workers, these relationships may form the basis of the practitioner’s employment (Kirkham et al. 2018), while nevertheless creating a complex web of overlapping responsibilities and obligations (Topp et al. 2018).

The smaller and more remote the community, the greater likelihood that there will be a blurring of clear professional boundaries (Halverson and Brownlee 2010). The web of overlapping relationships is also more likely to extend beyond that of patient—provider to include the patient’s and the professional’s family, friends and colleagues, and may include detailed knowledge of each others’ lives (Bourke et al. 2004). In addition to geography, it is recognized that the unique culture of each rural community is a strong contributor to the occurrence of overlapping relationships (Warner et al. 2005); where trust and engagement with a heath practitioner may be based on identifying the clinician as a member of that community (Kullnat 2014). Practitioner disengagement from overlapping relationships may even lead to a patient feeling rejected, thereby damaging trust and harming the therapeutic relationship (Nelson et al. 2007).

2. Practitioner Concerns Regarding the Management of Professional Boundaries

Rural health practitioners in Australia and elsewhere routinely report the inevitability and ubiquity of professional boundaries and their management as a pressing ethical concern (Endacott et al. 2006; Gardner et al. 2017). This appears particularly true for those working in the mental health field (Malone 2012), where it has been described as the most pressing ethical dilemma (Cates, Gunderson, and Keim 2012).

However, other empirical work has also highlighted that many practitioners hold the view that “extra”-professional contextual knowledge of a patient enhances patient support and provides holistic care (Brooks et al. 2012). For practitioners working with Aboriginal, Māori, Pasifika, and other First Nations peoples it may be an integral aspect of their professional practice (Smythe et al. 2018). The difficulty of balancing these competing demands is well recognized by Aboriginal Health Workers (Cosgrave et al. 2017), particularly where the community may expect continuous access (Cosgrave, Maple, and Hussain 2018). Aboriginal Health Workers amongst many others have identified the lack of support structures in place to assist with managing dual relationships (Conway et al. 2017).

3. Professional Guidelines for Managing Overlapping Relationships.

Within the Australian health context, formal codes of practice provide sparse guidance for the management of overlapping relationships. For instance, the Medical Board of Australia’s Code of Conduct (Medical Board of Australia 2020) provides at 10.2 that professional boundaries are “integral to a good doctor–patient relationship” and further that good medical practice involves “maintaining professional boundaries” and

… never using your professional position to establish or pursue a sexual, exploitative or other inappropriate relationship with anybody under your care. This includes those close to the patient … (Medical Board of Australia 2020)

Similarly, the Code of Ethics for Nurses in Australia provides mostly value statements with little prescriptive content for ethical practice (Australian College of Nursing and Australian Nursing Federation 2008).

The Australian Association of Social Workers’ Code of Ethics 2020 explicitly recognizes that providers may face unavoidable overlapping relationships and counsels the maintenance of appropriate boundaries and seeking external guidance as required (Australian Association of Social Workers 2020). In contrast to the health disciplines, this Association also provides an informal guideline aimed at assisting clinicians to recognize and manage dual relationships (Australian Association of Social Workers 2017).

4. Contextual Models for Managing Overlapping Relationships

Within the rural bioethics literature, a number of theorists have attempted to provide practical models to assist practitioners to evaluate ethically challenging situations involving dual relationships (Pugh 2007). A feature common to all models put forward is the requirement for critical self-reflection on the part of the practitioner (Brownlee et al. 2019). This generally involves the practitioner working through a checklist of questions to ensure they have considered the possible existence of a dual relationship as well as any risks of harm and other pertinent considerations (Pugh 2007). However, while the general approach is similar, the models differ substantially in their recommended treatment of multiple relationships.

In contrast to rule-based approaches, Campbell and Gordon recommend that clinicians should imagine the worst case scenario and then; (a) seek advice; (b) manage the relationship and (c); terminate the relationship as soon as possible (Campbell and Gordon 2003). Younggren and Gottleib propose a more nuanced inquiry into the nature of the dual relationship and any risks or harms that could be caused to the patient, the treating practitioner, and the therapeutic relationship, before warning that risks are ultimately unavoidable (Younggren and Gottleib 2004). Both Vig and Foglia (Vig and Foglia 2014), and Galbreath (Galbreath 2005) on the other hand adopt a more permissive and contextual approach; recognizing that there may be benefits to the overlapping relationship as well as harms (Vig and Foglia 2014; Galbreath 2005).

One concern that has been raised with adopting a flexible model is how a disciplinary body may retrospectively review the outcome of the practitioner’s decision, and their decision-making process (Pugh 2007); particularly as regulators will have access to more information than the practitioner at the time of the original decision (Freckelton and Flynn 2004). Australian regulatory bodies may be more likely to judge actions critically, resulting in legal and disciplinary sanctions (Spittal et al. 2016).

5. Philosophical Approaches to Managing Overlapping Relationships

Beyond the models outlined above, both virtue ethics (Crowden 2010) and the ethics of care (Unhjem et al. 2018) have been investigated as ethical frameworks to assist rural practitioners manage overlapping relationships. These philosophical approaches may assist practitioners navigate overlapping relationships by informing models of decision-making, and through contributing to a practitioner’s understanding of practical ethics. While utilitarianism’s pursuit of maximal benefit to the greatest number of people (Crowden 2010) and deontological ethics’ quest for good outcomes through adhering to overarching ethical principles (Crowden 2010) arguably require a more consistent and rigid approach to dual relationships (Crowden 2008b), virtue ethics and an ethics of care may permit clinicians a more realistic degree of flexibility in their decision-making (Crowden 2008b; Unhjem et al. 2018).

Within an Aristotelian virtue ethical framework (Oakley 2013), a person in a professional role should seek to develop virtues such as compassion and beneficence, and combine these with practical wisdom: thus allowing them to navigate ethically complex scenarios by drawing on their own resources and the practices and ethics of their chosen profession (Crowden 2008b). In Aristotelian terms, this is the practical virtue of phronesis (Arnold 2020), through which a professional will make nuanced decisions appropriate to the specific context (Crowden 2010). However the practitioner is not free to simply make a relativistic decision based on their own personal preference, they must act in a manner that is directed to advancing the realization of the patient’s goals (Crowden 2008a), in keeping with procedural and distributive justice, honouring the aims and virtues of their profession (Radden and Sadler 2008), and within the relevant regulative framework (Medical Board of Australia 2020).

Care ethics offers a more radical critique (Nordtug 2015) of the traditional western and patriarchal conceptualization of the ethical relationship that exists between a clinician and their patient (Unhjem et al. 2018). Beginning with a pre-existing ethical obligation on the practitioner to respond to the patient’s needs (Clifton-Soderstrom 2003), the decision to treat a patient in the context of multiple relationships requires critical reflection by the practitioner with respect to the particularity of the situation rather than reference to abstract norms (Nortvedt et al. 2011). So long as an overlapping relationship is consistent with furthering a patient’s well-being then boundary crossings are not only permissible but potentially ethically necessary (Nortvedt et al. 2011). However a stand-alone care ethics would seemingly necessitate a substantial zone of ethical ambiguity within which the practitioner would be required to operate thereby risking retrospective critique on the basis of ethical relativism (Pugh 2007; Elkin et al. 2012).

## Discussion

### Why Theory?

At the outset, it is worth briefly touching on the threshold question of the value of theory: particularly in the context of rural clinical practice. As noted, much of the bioethics literature is urban-centric and thus contains a set of assumptions in its worldview that may have limited applicability for rural and remote healthcare practice (Anderson, Pierce, and Crowden 2011). While health demographics across Australia, New Zealand, Canada, the United Kingdom, and other comparable countries indicate rural communities tend to have a higher morbidity and mortality rates (Disler, Glenister, and Wright 2020) with poorer access to services (Australian Institute of Health and Welfare 2019), theory may counterpoint this “deficit model” of healthcare and allow policy makers, administrators, and clinicians the opportunity to consider the resilience and strengths of the same communities (Bourke et al. 2010). Theory may likewise further empower professionals in their own practice (Combs and Freedman 2002) and assist them to make decisions that not only benefits their patients but also their own clinical practice and well-being (Simpson and McDonald 2017). An effective rural bioethics may require concurrent efforts at both the coalface of clinical practice and by researchers and administrators (Morley and Beatty 2008).

Guidance For Practitioners Working in Rural and Remote Communities

Given the evident dichotomy between (a) the lack of formal advice provided by the professional bodies and (b) the existence of various theoretical models, could further assistance be provided to practitioners working in rural and remote communities (Cook and Hoas 2008)? While cautioning against a generic approach (Gonyea et al. 2014), there is likely to be a tangible benefit in assisting practitioners to navigate situations such as whether to treat a friend (Zur 2005), play sport with a patient (Anderson, Pierce, and Crowden 2011), respond to an invitation to join a longstanding patient for dinner (Vig and Foglia 2014), negotiate the social fabric of a small town (Ringstad 2008), or to be able to contextualize their conduct to a regulatory body unfamiliar with the exigencies of practice in a particular location or setting (Scopelliti et al. 2004). The paradox faced by rural clinicians in having to impossibly partition their clinical and social worlds within a community (Gingerich et al. 2021) arguably provides a contextually informed rural ethics the opportunity to both support practitioners while more broadly making visible what is already a ethical reality (Simpson and McDonald 2017).

It is suggested that further guidance in navigating overlapping relationships could potentially come from peak bodies, colleges, employers, or universities during student rural rotations (Cook and Hoas 2019b; Gingerich et al. 2021). This could take form as guidelines, teaching scenarios, mentoring (Nickson et al. 2016), and orientation training amongst other approaches (Gillespie and Redivo 2012). This could be useful for isolated practitioners (Moran et al. 2009), and given the crucial role they play in rural locations generally, and particularly at Aboriginal Medical Services (Gilles et al. 2008) overseas trained doctors may find such guidance particularly valuable. For doctors working with Aboriginal patients, culturally safe management of overlapping relationships is likely to benefit both the patient and the practitioner (Deroy and Schütze 2019) and lead to increased retention rates and a sense of belonging (Dywili et al. 2012).

Overlapping relationships are often analysed through the “risk/benefit” lens which reinforces a deficit model of rural healthcare. This misconstrues the ends of healthcare, whose primary objective is to provide benefit.

Drawing on the models of decision-making together with the philosophical approaches reviewed above, one schema that could be used to help practitioners navigate overlapping relationships to provide beneficent healthcare and still flourish in their community is set out below:

### Acknowledge

- Overlapping relationships between one’s professional and social life are an inevitable part of working in rural and remote communities and can lead to valued therapeutic relationships and sustainable practice.
- These relationships are often dynamic in nature and may change and evolve over time. Likewise, a clinician’s approach to negotiating such relationships will alter depending on the nature of the relationship and the healthcare being provided.
- This schema aims to support practitioners to navigate dual relationships to ensure safe, fulfilling, and ethically sound therapeutic care.

### Recognize

- Are there any overlapping relationships with this patient or their family?
- What is the nature of those relationships?
- What are my own views and feelings in relation to this patient? To overlapping relationships generally?

### Assess

- What support, assistance, or treatment am I providing to this patient?
- What is the professional, community, and cultural context in which I am treating or working with this patient?
- Is there a risk that the therapeutic relationship could be disrupted or damaged by an overlapping relationship? Alternatively, is the overlapping relationship likely to benefit the therapeutic relationship?
- Are there risks to my own professional practice if the overlapping relationship continues?
- Is the overlapping relationship beneficial for my own professional and personal well-being?
- How does the overlapping relationship impact on other patients? On my workplace? On the community?
- Do the professional or ethical standards and values of my profession offer any guidance to how I should proceed?
- Are there any relevant codes of practice, rules, or laws applicable to this situation?
- Do I need to obtain advice from a colleague, mentor, supervisor, or professional body?
- Is this an urgent situation where I should provide care to the best of my ability regardless of the existence of a dual relationship that would normally stop me from treating the patient?

### Act

- Have I documented the existence of a relevant overlapping relationship?
- Do I need to discuss the overlapping relationship and impact on the therapeutic relationship with the patient? Obtain consent?
- Do I need to put any boundaries in place to allow me to maintain either the therapeutic relationship or the overlapping relationship?
- Do I need to appropriately refer the patient to another practitioner? To another service?
- Do I need to inform anyone else? My organization? Insurer? Professional body?

### Reflect

- How were my professional and personal values challenged by this situation?
- What worked well? What could I, my organization, or others have done differently?
- How might I handle a similar situation in the future?
- Is there a way I can share my experience with other practitioners to assist them to manage overlapping relationships in their own practice?
- Is this a situation where my own well-being requires me to debrief with others, particularly if there has been a challenging situation or poor outcome?

It is suggested that the “ARAAR” schema could be used as a “checklist” for practitioners encountering overlapping relationships and to help guide them in negotiating these relationships over time. The authors recognize that the schema may need to be adapted to different contexts and simply offer it as a framework for further development.

### Strengths and Limitations of This Review

From the outset it is acknowledged that utilizing a broad search strategy and having a single reviewer make decisions as to relevance has a significant subjective element, potentially introducing bias regarding the selection of papers ultimately included and any subsequent findings or conclusions.

However, for a bioethical review seeking to capture the heterogeneity of approaches to managing overlapping relationships, the authors felt that a wide-ranging literature review would capture publications not accessed by traditional search strategies and provide the benefit of introducing a broad range of perspectives that understand not only an academic perspective but also lay notions pertaining to dual relationships. For this reason, when checking for relevance to Australian practice using the criteria set out in the methodology, an inclusive approach was utilized that accepted papers that either had direct relevance due to similarities of professional practice environments, included theoretical approaches that were relevant, or provided commentary that was applicable to the Australian context. To ensure these criteria were met, disciplines such as social work were included alongside medical, nursing, psychological, and allied health perspectives while others further afield such as law and teaching were not despite the interest that these different perspectives might bring. It is hoped that by adopting such a strategy, that this review will provide readers with both an engaging and critical reading of current ethical approaches to overlapping boundaries in rural healthcare.

## Conclusion

When navigating overlapping relationships there is a diversity of approaches and different clinicians will make different decisions that are in keeping with their own values, those of their profession, the context of the organization, and the community within which they work (Nickel 2004). While the subjectivity of professional ethics makes it a challenging part of healthcare practice that demands ongoing critical reflection, there are also significant rewards to be had in its study and application (Austin et al. 2006).

This paper suggests that rather than adopt a strict formalistic approach to professional boundaries, an informed and reflective approach offers the practitioner, and the patient the best opportunity for an ethically sound therapeutic relationship that also allows for practitioners to make the most of working in rural and remote communities. Though perhaps radical in the abstract, the literature suggests that the existence and navigation of dual relationships is already part and parcel of sound ethical practice in “the bush,” and an everyday aspect of clinicians’ lives. While this approach may not fit within a traditional “urban-centric” view of practitioner–patient relationships, overlapping relationships may provide the basis for the provision of healthcare built on mutual recognition, acceptance, and understanding.
